# Novel protic ionic liquids-based phase change materials for high performance thermal energy storage systems

**DOI:** 10.1038/s41598-023-45549-7

**Published:** 2023-11-02

**Authors:** Masumeh Mokhtarpour, Ali Rostami, Hemayat Shekaari, Armin Zarghami, Saeid Faraji

**Affiliations:** 1 Department of Physical Chemistry, University of Tabriz, Tabriz, Iran; 2 Photonics and Nanocrystal Research Lab (PNRL), Faculty of Electrical and Computer Engineering, University of Tabriz, Tabriz, Iran

**Keywords:** Energy science and technology, Materials science

## Abstract

Phase change materials (PCMs) are an important class of innovative materials that considerably contribute to the effective use and conservation of solar energy and wasted heat in thermal energy storage systems (TES). The performance of TES can be improved by using environmentally friendly PCMs called ionic liquids (ILs) based on ethanolamines and fatty acids. The 2-hydroxyethylammonium, bis(2-hydroxyethyl)ammonium, and tris(2-hydroxyethyl)ammonium palmitate ILs, which function is in the temperature range of 30–100 °C and provide a safe and affordable capacity, are introduced in this study for the first time as PCMs. PCMs' chemical composition and microstructure were examined using fourier transformation infrared spectroscopy (FT-IR) and scanning electron microscopy (SEM), respectively. DSC was used to evaluate the ILs' latent heat of fusion and specific heat capacity, while TGA was used to establish their thermal stability. Finally, a home-made device with a PCMs (synthesized ILs) container cell and a commercial thermoelectric generator device to record the real-time voltage (V) was used to convert thermal energy into electrical energy.

## Introduction

Energy consumption has experienced unpredictable growth in recent decades due to technological advancements and improved living standards. To mitigate the adverse effects it generates, it is imperative to explore alternative energy generation methods rooted in renewable sources^1^. Due to some problems of traditional fuels, there is a global push to transition to cleaner and more sustainable energy sources, such as wind, solar, hydroelectric, and nuclear power, in order to address the environmental, economic, and social challenges associated with traditional energy sources. Applications of renewable energies help reduce greenhouse gas emissions, combat climate change, enhance energy security, and create sustainable and cleaner energy systems for the future. For many years, a well-known option has been thermal energy storage (TES), which comprises methods of energy storage in the form of sensible heat (resulting in a change in material temperature), the heat of chemical reaction, and latent heat associated with a phase shift. A phase change material (PCMs) is a substance that undergoes a phase transition (change in its physical state) from a solid to a liquid or from a liquid to a solid at a specific temperature, often referred to as its melting point or freezing point^2–4^. By using PCMs as energy storage, the energy supply and demand gap is reduced, energy distribution networks are made more efficient and reliable, and overall energy conservation is greatly increased^5–8^. The isothermal phase transition of PCMs also offers a broad variety of applications in proper temperature-sensitive systems, chemical reactors, the human body, and smart electrical devices^9–14^. Throughout the development of PCMs, research has been conducted on a wide range of material classes, including chemical compounds (such as fatty acids and paraffin), mineral compounds (salt hydrates and salts), and even polymeric materials (such as PEG)^15,16^.

A broad electrochemical stability window, strong ionic conductivities, low vapor pressures, thermal stability, and lack of flammability are just a few of the notable advantages of the new family of materials known as ionic liquids (ILs). Due to their exceptional qualities, ILs have found extensive usage as "green" organic solvents, electrolytes in double-layer capacitors, fuel cells, batteries, and dye-sensitive solar cells. In the last ten years, several fundamental investigations on the thermodynamic properties of ILs for application as thermal storage medium and heat transfer fluids have been conducted^17–19^.

However, unlike paraffin, which is made from petroleum, fatty acids may be derived from living resources without the need of fossil fuels^20^. They have several advantageous qualities, including as affordability, non-toxicity, and corrosion resistance. Additionally, they frequently exhibit favorable phase transition temperatures, great ability to store latent heat, no supercooling or phase separation, and strong thermal and chemical stability^21–23^.

As researchers looked for more environmentally friendly chemical processes, the field of ILs was developing and fast increasing as the globe struggled with the terrible effects of the carbon-based energy sector^24–26^. In light of the fact that many ILs have low flammability, volatility, and corrosivity, which are drawbacks of conventional PCM materials, it has recently been obvious that these features are advantageous for PCMs^27^. A number of ILs have been used as PCMs for TES applications^28–32^. The value of ILs based on hydroxyethyl ammonium is considerable since they are also used as thermal energy storage devices^33–35^.

Solar power plants are one of the renewable energy power plants that the international community considers as national sources of high-capacity power generating. Solar panels have the potential to be diminished or even destroyed in the absence of sunlight. PCMs that have performed well have been utilized to solve this issue. In order to provide electrical compensation, PCMs will evaluate the solar panel's thermal performance with a new design. In this study, we investigate the state-of-the-art PCMs for TES applications in solar cells to produce electricity, as well as current efforts to develop innovative PCMs with enhanced performance and security. TES materials were created using PCMs based on 2-hydroxyethylammonium palmitate, bis(2-hydroxyethyl)ammonium palmitate, and tris(2-hydroxyethyl)ammonium palmitate. PCMs' microstructure and chemical makeup were examined using Fourier transformation infrared spectroscopy (FT-IR) and scanning electron microscopy (SEM), respectively. DSC and TGA studies were used to calculate the prepared PCMs' latent heat of fusion and thermal stability. Finally, a home-made instrument with a PCM container cell and a commercial thermoelectric generator device to record the real-time voltage (V) was used to execute the thermal-to-electric energy conversion.

## Experimental measurements

### Chemicals

The following sources provided the materials for this study: Mono, di, and triethanolamine from Shazand Petrochemical Co. had a purity greater to 99%, and palmitic acid from Merck Co. Ethanolamines were simply neutralized by palmitic acid in an acid–base reaction to produce the ionic liquids (2-hydroxyethylammonium palmitate), bis(2-hydroxyethyl)ammonium palmitate, and tris(2-hydroxyethyl)ammonium palmitate. They were not further purified before usage.

### Synthesize of ionic liquid as phase change materials (PCMs)

**Figure 1:**
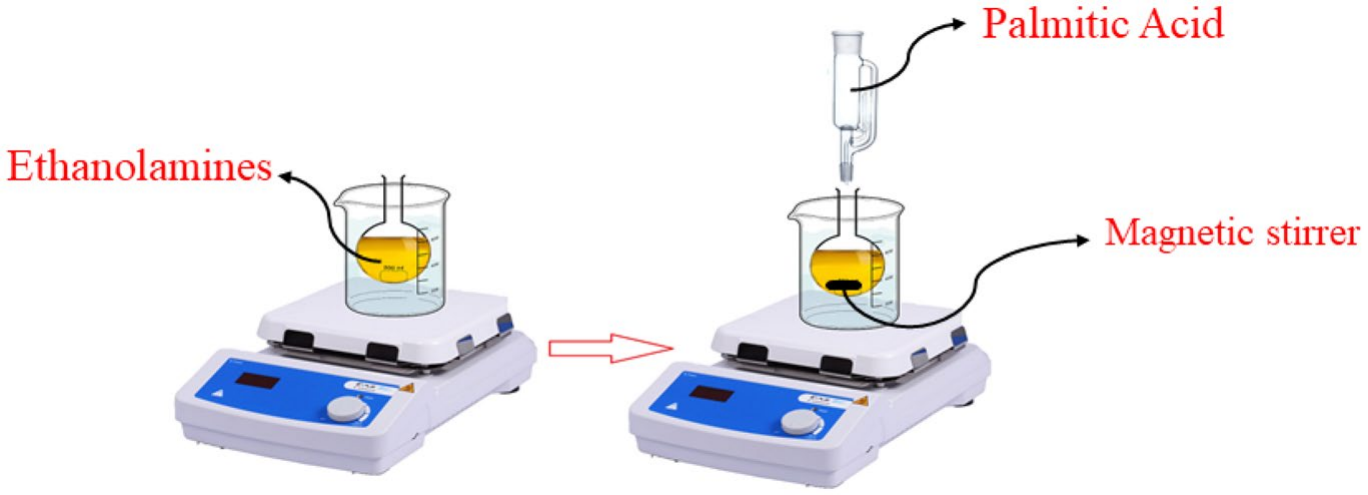
Schematic of the synthesized ionic liquids as phase change materials (PCMs).

First, the ethanolamines were introduced to a glass flask with three necks and a reflux condenser. The magnetic stirrer was used to agitate the flask while the molten palmitic acid was added dropwise. Stirring was kept up for 24 h at room temperature to finish the reaction. All compounds' specifications are shown in Table 1. Schematic of the synthesized ionic liquids as phase change materials (PCMs) is shown in Fig. 1^36^.

**Table 1:** Information of used chemicals.

Chemicals	Source	CAS no.	Molar mass (g∙mol^−1^)	Mass percent (purity)
Monoethanolamine (MEA)	Shazand petrochem	141-43-5	61.08	> 99
Diethanolamine (DEA)	Shazand petrochem	111-42-2	105.14	> 99
Triethanolamine (TEA)	Shazand petrochem	102-71-6	149.19	> 99
Palmitic acid	Merck	57-10-3	256.4	≥ 98
2-hydroxyethylammonium palmitate [HEA]Pal	Synthesized in our lab	–	317.48	> 98
bis(2-hydroxyethyl)ammonium palmitate [DHEA]Pal	Synthesized in our lab	–	361.54	> 98
Tris(2-hydroxyethyl)ammonium palmitate [THEA]Pal	Synthesized in our lab	–	405.59	> 98

The suppliers were provided the purities of the used components.

### Chemical characterization

FT-IR Transmittance Spectra of the PCMs, [HEA]Pal, [DHEA]Pal, and [THEA]Pal via KBr have been recorded using Bruker (Tensor 270-KBr). Also, to examine the microstructure and morphology of produced PCMs, a device scanning electronic microscope (TESCAN, MIRA3 FEG-SEM) was utilized.

### Thermal characterization

To ascertain the thermophysical characteristics of the newly developed PCMs [HEA]Pal, [DHEA]Pal, and [THEA]Pal, produced ILs were measured calorimetrically. With a thermal investigation utilizing a differential scanning calorimeter (Netzsch DSC-200 F3) up to 373 K, the heat capacities and heat of fusion were characterized. To achieve this, heating rates of 10 K/min were used throughout the thermal stages. The samples were heated to 373 K in this regard. The heating stage allowed for the determination of the PCMs' thermal characteristics, including heat capacity and heat of fusion. At a rate of 10 K/min, the PCMs' specific heat capacity were measured between 313.15 and 348.15 K. PCM sample weights were measured using an electronic balance with a precision of ± 10^−3^ g. The DSC device has a sensitivity of 10^–5^ µV·mW^−1^.

In addition, a thermal analysis equipment (METTLER TOLEDO, TGASDTA 851e) was used to record the thermogravimetry study of the synthesized PCMs, [HEA]Pal, [DHEA]Pal, and [THEA]Pal in order to assess their thermal stability across a range of 50–700 °C under N_2_ (30 mL min^−1^).

### Thermal-to-electric energy conversion and storage

The creation of system configurations, simulation models, prototype trials, and electrical and thermal performance assessment have dominated research into solar cells-PCM systems. The possibility of enhancing the PV module's ability to generate energy through the use of a PCM was examined in this study through an experiment. A home-made instrument containing a PCM container cell and a commercial thermoelectric generator device to record the real-time voltage (V) was used to convert heat energy into electrical energy. We employed a vacuum cup filled with a heat sink as a heat source and a sample that absorbed and collected heat from a simulated thermal source as a cold source.

## Results and discussion

Due to their superior heat transfer characteristics, non-volatility, non-flammability, and high chemical and thermal stability, ionic liquids (ILs) based on monoethanolamine, diethanolamine, triethanolamine with palmitic acid are good PCM candidates^37^. Additionally, a wide variety of cations and anions makes it possible to construct ILs with the ideal characteristics, notably melting point, for the desired usage. Using the 2-hydroxyethylammonium [HEA]Pal, bis(2-hydroxyethyl)ammonium [DHEA]Pal, and tris(2-hydroxyethyl)ammonium palmitate [THEA]Pal ILs as a base, we describe a unique PCM here.

### Characterization of the synthesized PCMs

#### FT-IR spectrum of the synthesized PCMs

**Figure 2:**
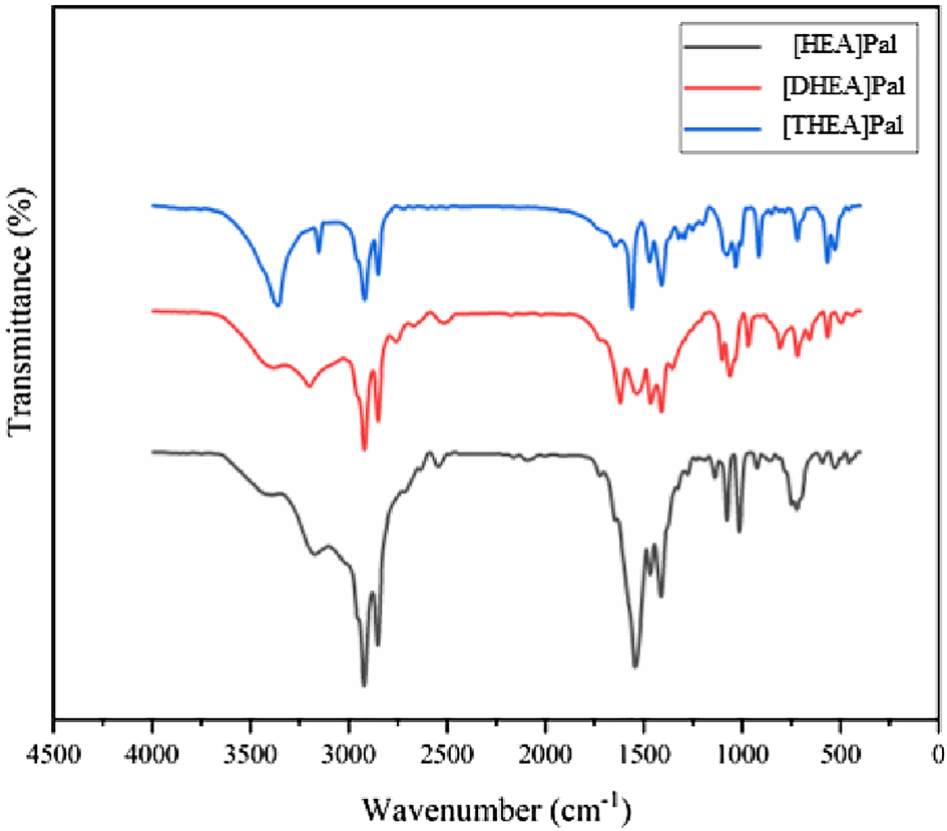
The FT-IR spectra of the [HEA]Pal, [DHEA]Pal and [THEA]Pal.

The FT-IR spectra of the synthesized ILs [HEA]Pal, [DHEA]Pal and [THEA]Pal are depicted in Fig. 2. The FT-IR index peaks for palmitic acid are as follows: 549, 685, 723, 939, 1261, 1299, 1422, 1464, 1695, 2848, 2913 and 2950^38^. The FT-IR index peaks for ILs are as follows. FT-IR (KBr, cm^−1^) for [HEA]Pal IL: 528.80, 721.22, 1013.48, 1076.48, 1138.10, 1411.44, 1466.81, 1542.72, 2851.96, 2921.53, 3170.78, and 3384. FT-IR (KBr, cm^−1^) for [DHEA]Pal IL: 566.04, 718.90, 806.16, 966.61, 1061.17, 1102.76, 1409.71, 1464.57, 1536.04, 1620.99, 2850.74, 2920.08, 3199.13, and 3380.40. FT-IR (KBr, cm^−1^) for [THEA]Pal IL: 529.44, 566.62, 719.30, 915.44, 1031.06, 1075.54, 1294.29, 1408.57, 1470.18, 1561.81, 2850.64, 2918.83, 3151.99, and 3359.21. As seen in Fig. 2, the ILs displayed peaks in the same locations as the characteristic peaks of monoethanolamine, diethanolamine, triethanolamine and palmitic acid, which is ascribed to the [HEA]Pal, [DHEA]Pal and [THEA]Pal having the same molecular structure. The substance's characteristics may be accurately predicted by looking at the COO^−^ absorption peaks, which are located at roughly 528 and 720 cm^−1^. Additionally, the long-chain alkyl group is reflected by the strong distinctive peaks at 2850 and 2920 cm^−1^, which are associated with the symmetric and anti-symmetric detention vibration peaks of CH_2_, respectively. Peaks at roughly 1464–1470 cm^−1^ are used to represent the in-plane bending, symmetric, and out-of-plane bending vibrations of the fatty acid carboxyl COOH, respectively. Peaks at approximately 1075 cm^−1^ are used to represent the anti-symmetric stretching vibration peaks of CH_3_, where they appear as two faint peaks^39^. In all ILs, there is a peak that corresponds to N–H at about 3359–3384 cm^−1^. According to the index peaks of palmitic acid and the synthesized ILs, it can be concluded that the ILa were correctly synthesized and the additional peaks in the ILs were used for the raw materials of ethanolamines.

#### SEM imaging of the synthesized PCMs

**Figure 3:**
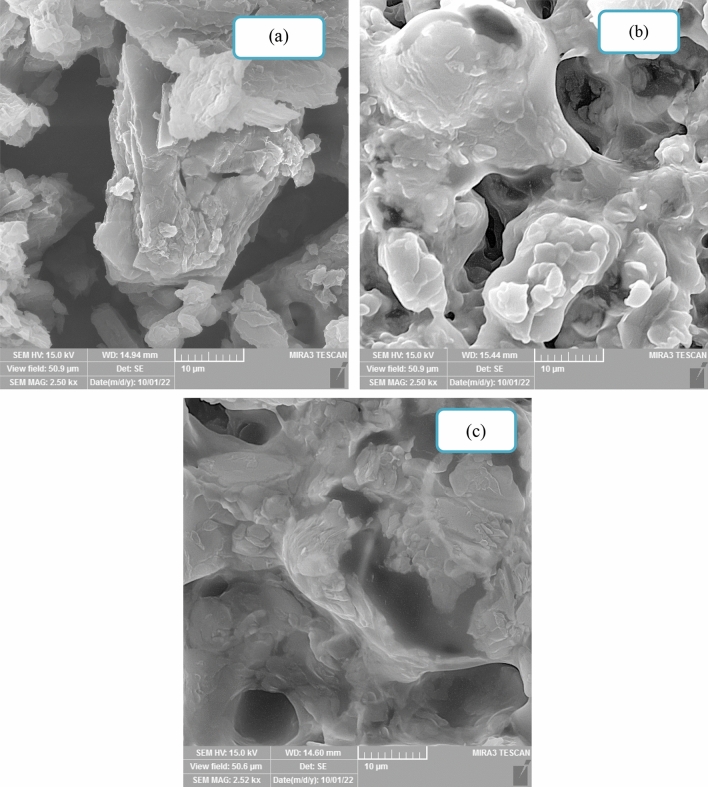
The SEM images of the PCMs; (**a**) [HEA]Pal, (**b**) [DHEA]Pal and (**c**) [THEA]Pal.

The SEM images of the synthesized PCMs are shown in Fig. 3. The ILs have a rough surface and a layered structure, as seen in this image^40^. As seen in Fig. 3, monoethanolamine, diethanolamine, and triethanolamine all responded well to the palmitic acid. The mechanical strength of ILs is increased, and they stop the leakage of monoethanolamine, diethanolamine, and triethanolamine as a result of interactions between the ionic force in the network structure of the generated ILs and the surface tension forces of fatty acid^39,40^. Another observation from Fig. 3 is that [HEA]Pal is smaller size than [DHEA]Pal and [THEA]Pal and has more homogeneous microstructures. The ILs are all layered and in the form of laminates, which have a greater surface ratio than the raw materials and need more energy to melt and solidify, as can be seen from the SEM photos.

### Thermal analyses of the synthesized PCMs

#### Differential scanning calorimetry (DSC) of the synthesized PCMs

**Figure 4:**
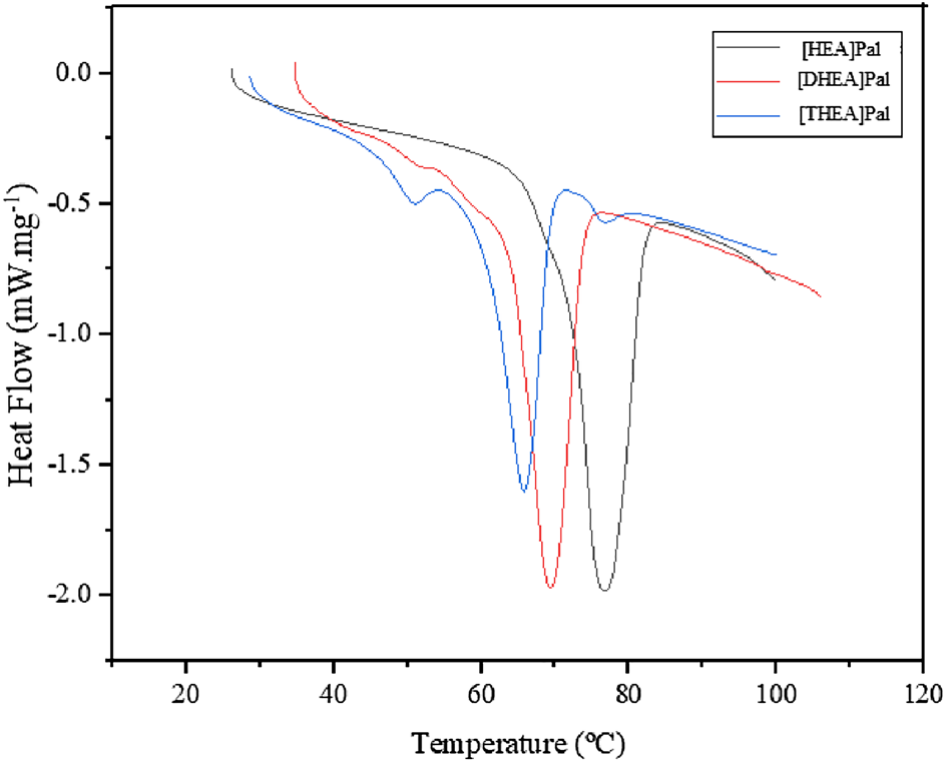
The DSC analysis of the PCMs.

The calorimetry data for the synthesized PCMs are displayed in Fig. 4 and given in Table 2. According to the DSC graphs, Fig. 4 depicts the heating curves (exothermic up) for the ILs [HEA]Pal, [DHEA]Pal, and [THEA]Pal. The melting points for the ILs [HEA]Pal, [DHEA]Pal, and [THEA]Pal are 349.43 K, 342.58 K, and 338.92 K, respectively, as indicated in Table 2. According to the findings, the IL [HEA]Pal requires a stronger structure and more energy to melt than the ILs [DHEA]Pal and [THEA]Pal.

**Table 2:** DSC and TGA data of the synthesized PCMs.

Chemicals	Melting point (K)	Latent heat (kJ·kg^−1^)	Thermal stability or residue amount (%) up to 150 °C
Palmitic acid	337.18^43^	185.4^43^	–
	332.05^44^	189.6^44^	–
	339.05^45^	218.4^45^	–
[HEA]Pal	349.43	158.9	97
[DHEA]Pal	342.58	139.8	99
[THEA]Pal	338.92	123.2	100

The characteristics of the produced ILs during phase transitions are very comparable to the composition of palmitic acid. The melting latent heat for the [HEA]Pal, [DHEA]Pal, and [THEA]Pal are determined to be 158.9 kJ kg^−1^, 139.8 kJ kg^−1^, and 123.2 kJ kg^−1^, respectively, according to Table 2. The ionic forces in the cation and anion create the latent heat of this kind of ILs when acidic H is transferred from palmitic acid to the NH_2_ group of ethanolamines.

**Figure 5:**
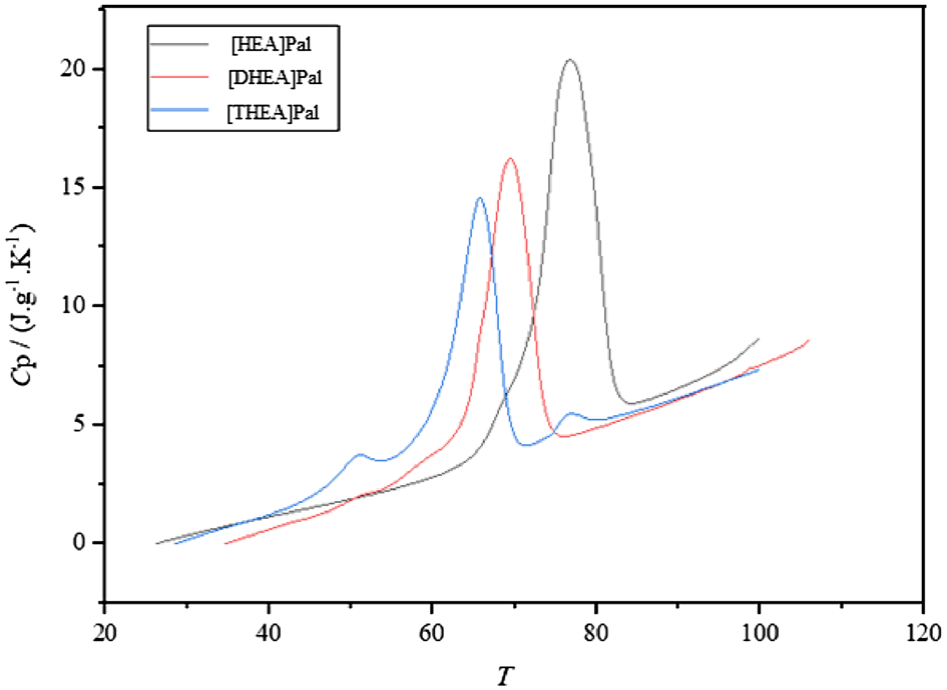
The specific heat capacity of the PCMs.

The *C*p values of the synthesized ILs at *T* = 313.15 to 373.15 K were calculated using the DSC data and are shown in Fig. 5 and Table 3 respectively. Despite the fact that all ILs contain the same anion—palmitate—there are variations in the examined ILs' specific heat capacities because of the various cations at play. Due to a larger ionic force between the cation and anion in IL [HEA]Pal than in the other two ILs, the *C*p values are higher.

**Table 3:** The heat capacities of the [HEA]Pal, [DHEA]Pal and [THEA]Pal at the melting point and at different temperatures.

*T *(K)	[HEA]Pal	[DHEA]Pal	[THEA]Pal
313.15	1.136	0.601	1.222
318.15	1.505	1.103	1.975
323.15	1.875	1.183	3.520
328.15	2.292	2.495	3.605
333.15	2.803	3.783	5.729
338.15	3.758	7.093	13.805
338.92	–	–	14.594
342.58	–	16.243	–
343.15	7.001	15.973	4.487
348.15	18.021	4.641	4.851
349.43	20.394	–	–
353.15	13.995	4.883	5.216
358.15	5.952	5.463	5.618
363.15	6.553	6.080	6.152
368.15	7.367	6.743	6.758
373.15	8.671	7.535	7.349

#### Thermogravimetry analyses (TGA) of the synthesized PCMs

**Figure 6:**
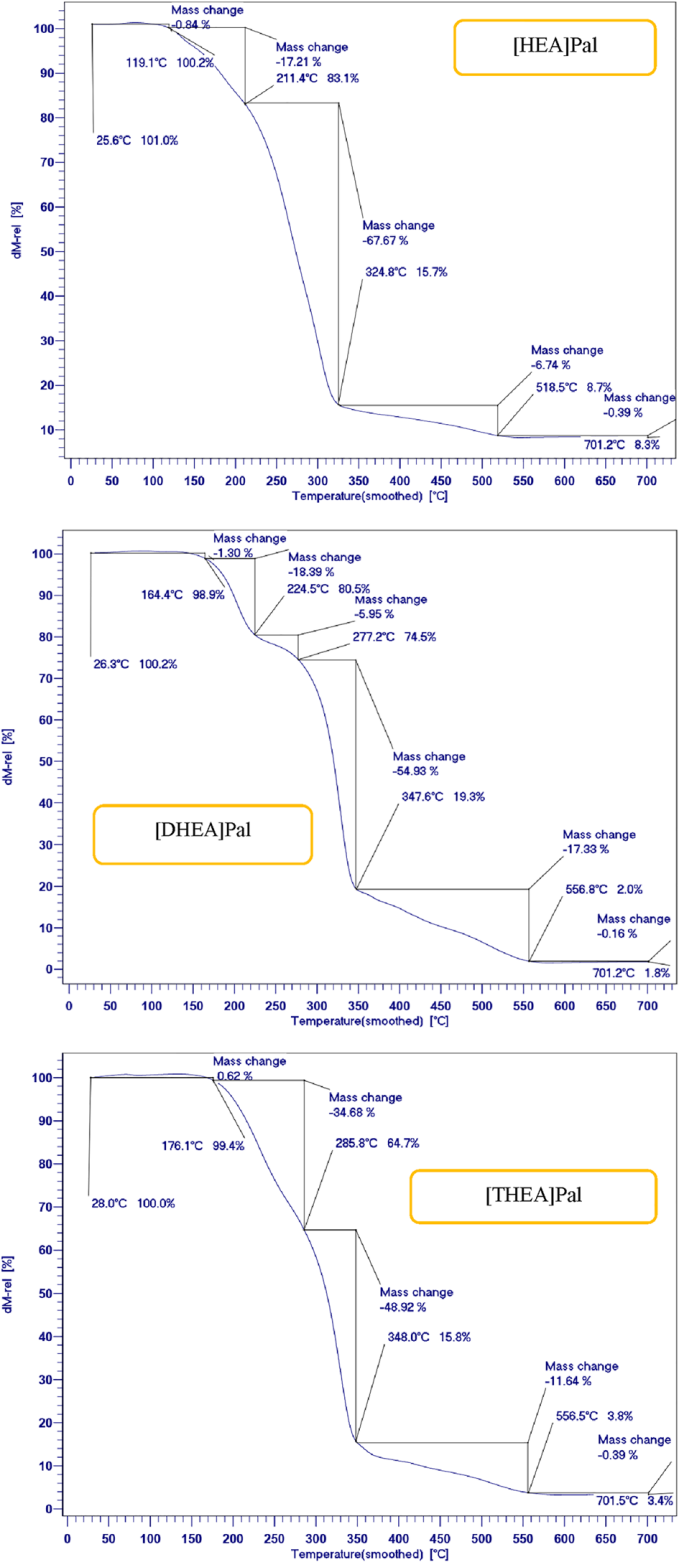
The quasistatic TGA of the PCMs.

Figure 6 displays the TGA and DTG curves for the ILs [HEA]Pal, [DHEA]Pal, and [THEA]Pal. The amount of charred residue at 700 °C and the temperature at which the most weight is lost are shown in Table 2. The three-step heat deterioration mechanisms are shown in Fig. 6. The weight loss of the [HEA]Pal is considerably smaller throughout three-step thermal degradation processes than that of the [DHEA]Pal and [THEA]Pal. As seen in Fig. 6, the first step, which corresponds to the release of water molecules adsorbed in the network of ILs, occurs between 20 and 100 °C. The second stage occurs between 100 and 220 °C, where the ILs' molecular chains experience a 45% thermal degradation. Users of solar thermal energy storage are advised to utilize the synthesized ILs in this study. Given that solar cell systems and solar thermal storage are capable of operating at temperatures of up to 150 °C, the synthesized ILs exhibit above 97% thermal stability at this temperature, indicating the need for appropriate PCMs. The results shown in Fig. 6 show that [HEA]Pal, [DHEA]Pal, and [THEA]Pal have respective thermal stabilities of 97, 99, and 100%. According to the analysis of thermal stability, it can be understood that the synthesized ILs showed remarkable thermal stability compared to the used palmitic acid and ethanolamines and are stable at higher temperatures^38,41,42^.

### Real-time voltage (V) results

**Figure 7:**
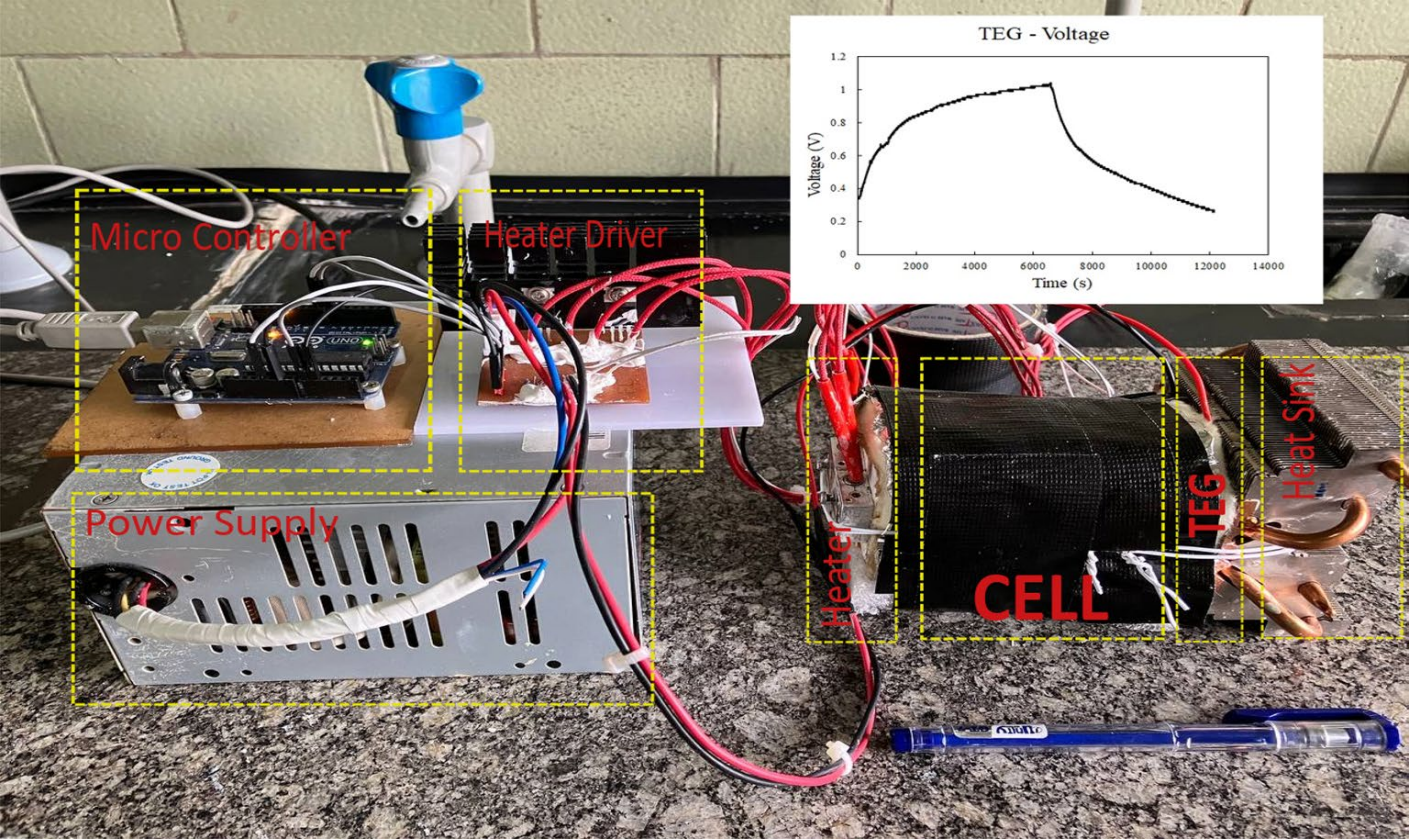
Schematic of the instrument.

**Figure 8:**
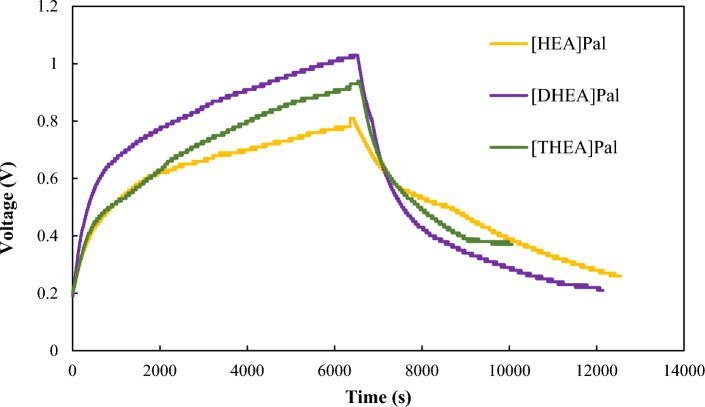
Voltage versus time in TEG system for PCMs.

A homemade instrument was performed for the thermal-to-electric energy conversion with a commercial thermoelectric generator device to record the real-time voltage (V). Schematic of the instrument is shown in Fig. 7. In this regard, the electrical voltage output of cell containing ILs as a new generation of PCMs is represented in Fig. 8, which indicated that electrical voltage of PCM based on [HEA]Pal is higher than [DHEA]Pal and [THEA]Pal.

It is found that a PCM as a practical storage medium may achieve a 20% greater total day electrical output per unit storage volume than liquid water in a full-storage approach where electrical energy generation from the PCM is offset to meet the weekday evening peak in demand. The isothermal operation of the PCM during phase-change enables a lower diurnal storage temperature variance and a greater energy conversion efficiency from the solar collector array.

As a result of this research work, PCM performance for thermal energy storage applications will be enhanced by the employment of a synthesized ILs with noteworthy features. According to FT-IR data, chemical processes take place during the creation of ILs. The ILs were also discovered by SEM to be in the form of layered, homogenous microcrystals, and by heating them, a lot of energy is released in the solid–liquid process because of the higher number of layers.

Utilizing novel materials with high latent heats of fusion, heat capacities, and thermal stability will enable the introduction of thermal energy storage applications. In this regard, the synthesized ILa as PCMs described in this study have greater latent fusion energies up to about 158.9 kJ kg^−1^, large heat capacities, and high thermal stabilities up to 97%. The main takeaway from this study is the effectiveness of PCMs with excellent performance for solar cell thermal energy storage. In this manner, solar cells lose heat up to 150 °C. This unused heat may be turned into energy by using PCMs. It is conceivable to eliminate the need for power at night by continually releasing the latent heat of fusion of these PCMs, which may be accomplished by storing the wasted heat in PCMs when there is no sunlight.

## Conclusions

There is a lot of potential for significant, environmentally friendly, and long-lasting effect with the newly developed use of ionic liquids for renewable thermal energy storage. The development of phase transition materials based on palmitic acid and ethanolamine ([HEA]Pal, [DHEA]Pal, and [THEA]Pal) is presented. DSC studies revealed that the PCMs' melting point was in the range of 30–100 °C. For the [HEA]Pal, [DHEA]Pal, and [THEA]Pal, the latent heat of fusion was calculated to be 158.9, 139.8, and 123.2 kJ kg^−1^, respectively. It is clear that among them, the [THEA]Pal has the maximum thermal stability, indicating that it is a good PCM up to 150 °C. The increased size of the cation, which results in the production of strong ionic interactions in the structure of this IL, may be the cause of the [THEA]Pal's improved thermal characteristics. Additionally, as seen by the shape of ILs, [HEA]Pal becomes compressed and coiled around itself as the cation size decreases. More energy was required to disassemble its structure. The [HEA]Pal has higher *C*p values than the other two ILs, indicating a larger storage capacity.

## Data Availability

All data generated or analyzed during this study are included in this published article.
